# *Psammolestes tertius* Lent & Jurberg, 1965 (Hemiptera, Reduviidae, Triatominae): first report in Sergipe State, Brazil

**DOI:** 10.1590/0037-8682-0708-2020

**Published:** 2021-03-22

**Authors:** Jader de Oliveira, João Aristeu da Rosa, Felipe Mendes Fontes, David Campos Andrade, Rubens Riscala Madi, Cláudia Moura de Melo

**Affiliations:** 1 Universidade de São Paulo, Faculdade de Saúde Pública, São Paulo, SP, Brasil.; 2 Universidade Estadual Paulista Júlio de Mesquita Filho, Faculdade de Ciências Farmacêuticas, Departamento de Ciências Biológicas, Araraquara, SP, Brasil.; 3 Universidade Tiradentes, Programa de Pós-Graduação em Saúde e Ambiente, Aracaju, SE, Brasil.; 4 Instituto de Tecnologia e Pesquisa, Laboratório de Biologia Tropical, Aracaju, SE, Brasil.; 5 Instituto de Tecnologia e Pesquisa, Laboratório de Doenças Infecciosas e Parasitárias, Aracaju, SE, Brasil.

**Keywords:** Biogeography, Caatinga, Geographical distribution, Kissing bugs

## Abstract

**INTRODUCTION:**

This study reports the first occurrence of *Psammolestes tertius* (Hemiptera, Reduviidae, Triatominae) in the state of Sergipe, Brazil.

**METHODS:**

In 2020, 95 specimens were collected from the municipality of Porto da Folha, Sergipe, Brazil.

**RESULTS:**

This finding expands the geographical distribution of the species from 15 states in Brazil to 16 and increases the biodiversity of triatomines in the state of Sergipe.

**CONCLUSIONS:**

The presence of *P. tertius* in the state of Sergipe demonstrated a wider distribution of this species in northeastern Brazil.

Described by Jeannel (1919)^1^, the subfamily Triatominae is recognized by mandatory hematophagy. Various species are important vectors of *Trypanosoma cruzi* (Chagas 1909) (Kinetoplastida, Trypanosomatidae), the etiologic agent of Chagas disease, while others are of little importance from an epidemiological point of view, especially those with low adaptability to artificial ecotopes^2^. Currently, the subfamily Triatominae contains more than 150 species, grouped into five tribes and 18 genera^3^ ^,^ ^4^. 

The genus *Psammolestes* Bergroth, 1911 is a monophyletic group comprising only three species: *P. arthuri* Pinto, 1926; *P. coreodes* Bergroth, 1911; and *P. tertius* Lent and Jurberg, 1965^5^. Despite the genus *Psammolestes* having a close association with birds, they are not the only source of food for these triatomines in nature. For other groups, for example mammals, have already been found inhabiting the nests of birds^6^ ^,^ ^7^.

Species of the genus *Psammolestes* have been found in nests of several bird families: Dendrocolaptidae (woodcreepers), Troglodytidae (house wren and long-billed wren), Furnariidae (rufous-fronted thornbird and rufous hornero), Icteridae (troupial, oropendola, yellow oriole, and yellow-rumped)^8^ ^-^ ^13^.

*Psammolestes tertius* is distributed in all geographic regions of Brazil: the Southeast (Minas Gerais and São Paulo), Midwest (Goiás and Mato Grosso), North (Pará and Tocantins), Northeast (Alagoas, Bahia, Ceará, Maranhão, Paraíba, Pernambuco, Piauí, and Rio Grande do Norte), and South (Paraná), and in Peru (San Martín)^6^ ^,^ ^8^ ^,^ ^14^ ^,^ ^15^. The comparison of two different populations of *P. tertius* showed associations with different species of Furnariidae birds from the states of Ceará and Minas Gerais. The use of different RAPD markers has demonstrated intraspecific variability, possibly related to environmental changes characteristic of Caatinga and Cerrado^16^. However, this variability was not corroborated by a cytogenetic comparison of two populations (Bahia and Ceará) carried out by Oliveira et al.^6^.

In the Sergipe state, northeastern Brazil, all the following species have already been reported: *Panstrongylus lutzi* Neiva and Pinto, 1926; *P. megistus* Burmeister, 1835; *P. geniculatus* Latreille, 1811; *Rhodnius neglectus* Lent, 1954; *R. zeledoni* Stal, 1859; *Triatoma brasiliensis* Neiva, 1911; *T. melanocephala* Neiva and Pinto, 1923; *T. pseudomaculata* Corrêa and Spínola, 1964; *T. petrocchiae* Pinto and Barreto 1925; *T. rubrofasciata* De Geer, 1773; and *T. tibiamaculata* Pinto, 1926^14^ ^,^ ^17^ ^-^ ^21^. Although Sergipe borders Bahia and Alagoas, which are states where *P. tertius* occurs, this is the first record of this species in that state. 

Sergipe has an area of approximately 21,926.908 km^2^, corresponding to 0.26% of the national territory, and an estimated population of 2.06 million. It comprises 75 municipalities distributed in three climatic regions: semi-arid, agreste, and humid coastal^22^ ^,^ ^23^. The municipality where insects were collected is Porto da Folha (9° 54′ 34″ S; 37° 16′ 41″ W), which has an area of 876.67 km² and is 156 km from the state capital, Aracaju (Figure 1). 

**FIGURE 1:**
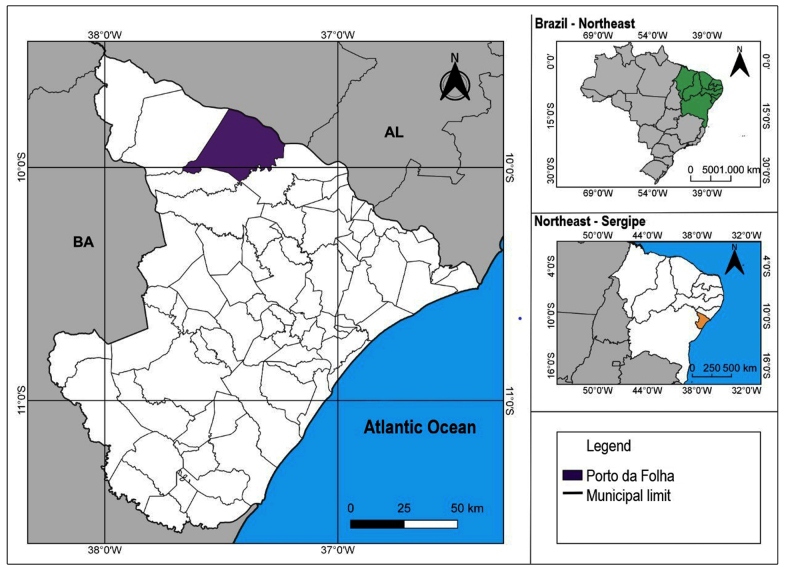
Geographical localization of the Sergipe state (Orange), highlighting Porto da Folha, Sergipe (purple) and the Brazilian northeast in green.

New records: Brazil, Sergipe, municipality of Porto da Folha, within the Getirana village. Three adults and 20 nymphs were collected from nests of birds popularly known as casaca-de-couro [*Pseudoseisura cristata* (Spix, 1824)]. The 3 adults (2 females and 1 male) were collected alive at 17:45 h local time on January 13, 2020 (first record). The second collection included 9 males, 10 females, and 55 nymphs of the species *P. tertius*; these and other insects were associated with nests of casaca-de-couro birds [*P. cristata* located on *Mimosa tenuiflora* trees (Willd.) Poiret]. The females and males were collected alive at 15:35 h local time on January 14, 2020 (last record).

Both specimens were identified as *P. tertius* based on their head characteristics (Figure 2). Since their heads are slightly longer than broad, at the level of the eyes, with a moderate slope behind the ocelli, the pronotal anterolateral angles are very short and obtuse. On the other hand, *P. coreodes* has a head with a length equal to or slightly shorter than the width at the level of the eyes, with a sharp slope behind the ocelli, and the anterolateral angles of the pronotum are cuspidal². The analyzed specimens were deposited in the José Maria Soares Barata entomological collection in batch format with the voucher CEJMSB 861.

**FIGURE 2:**
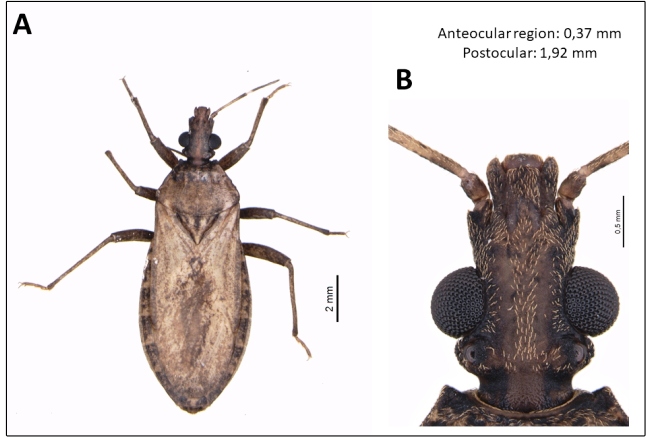
*Psammolestes tertius*. **(A)** Female. **(B)** Detail of head.

The genus *Psammolestes* comprises three species and seems to have specialized in exploiting bird nest microhabitats, mainly in the family Furnariidae^15^. These species are distributed in South America and have clear ecological niche differences. A recent study based on distributional data predicted the potential geographical distribution of the three *Psammolestes* species using ecological niche modeling. The models suggest that *P. arthuri* are distributed in warm and humid areas, *P. coreodes* occupy the driest and coldest areas, and *P. tertius* have intermediate climatological limits and occur at the highest altitudes. For *P. tertius*, this potential area includes the Rio Grande do Norte state^24^. Therefore, this record of a new point of occurrence of *P. tertius* is very important for future work and reinforces the results obtained in a theoretical model by Gurgel-Gonçalves and Silva^25^. 

In addition to the probable influence of climatic zones and altitude variations in the distribution of *P. tertius*, another potential means of dispersion to other regions is that its eggs are secreted with adhesive substances during oviposition and can adhere to substrates such as bird feathers. This association with birds, mainly as vehicles of migratory dispersion, may eventually favor the passive capture of these species of triatomines in new areas of occurrence^25^ ^,^ ^26^. It is worth highlighting that the knowledge about triatomine species that have these passive dispersion mechanisms, combined with the results obtained from the theoretical model of Gurgel-Gonçalves and Silva^25^, is fundamental for future studies on new species and to auxiliary entomology programs in Brazil.
